# Myocardial Metabolism in Heart Failure with Preserved Ejection Fraction

**DOI:** 10.3390/jcm13051195

**Published:** 2024-02-20

**Authors:** John Aaron Henry, Liam S. Couch, Oliver J. Rider

**Affiliations:** 1 Oxford Centre for Clinical Magnetic Resonance Research, Division of Cardiovascular Medicine, Radcliffe Department of Medicine, University of Oxford, Oxford OX3 9DU, UKoliver.rider@cardiov.ox.ac.uk (O.J.R.); 2 Department of Cardiology, Jersey General Hospital, Gloucester Street, St. Helier JE1 3QS, Jersey, UK

**Keywords:** HFpEF, heart failure, cardiac metabolism, substrate utilisation, energy production

## Abstract

Heart failure with preserved ejection fraction (HFpEF) is increasingly prevalent and now accounts for half of all heart failure cases. This rise is largely attributed to growing rates of obesity, hypertension, and diabetes. Despite its prevalence, the pathophysiological mechanisms of HFpEF are not fully understood. The heart, being the most energy-demanding organ, appears to have a compromised bioenergetic capacity in heart failure, affecting all phenotypes and aetiologies. While metabolic disturbances in heart failure with reduced ejection fraction (HFrEF) have been extensively studied, similar insights into HFpEF are limited. This review collates evidence from both animal and human studies, highlighting metabolic dysregulations associated with HFpEF and its risk factors, such as obesity, hypertension, and diabetes. We discuss how changes in substrate utilisation, oxidative phosphorylation, and energy transport contribute to HFpEF. By delving into these pathological shifts in myocardial energy production, we aim to reveal novel therapeutic opportunities. Potential strategies include modulating energy substrates, improving metabolic efficiency, and enhancing critical metabolic pathways. Understanding these aspects could be key to developing more effective treatments for HFpEF.

## 1. Introduction

Heart failure with preserved ejection fraction (HFpEF) accounts for half of all heart failure diagnoses and is associated with similar morbidity and mortality to heart failure with reduced ejection fraction (HFrEF) [1]. Until recently, there were no treatments which were shown to have a prognostic benefit in HFpEF [2,3]. It is growing in prevalence, owing largely to increases in risk factors such as obesity, hypertension, and diabetes within an ageing population [1]. Despite this, there is a lack of clear understanding of the pathophysiological processes at play in this condition. Moreover, the possibility of distinct HFpEF phenotypes has emerged, including cardiometabolic, elderly hypertensive, right heart/pulmonary dysfunction, and left atrial myopathy. Broadly speaking, the condition is characterised by impaired cardiac relaxation and diastolic dysfunction, resulting in high filling pressures. The factors leading to this impaired relaxation are complex but are likely to be related to passive factors such as increased myocardial fibrosis and inherent cardiomyocyte stiffness, and active factors involved with ATP-dependent relaxation. This review focuses predominantly on these active mechanisms.

The heart is a highly energy-demanding organ, consuming more energy than any other organ at around 6 kg of ATP per day [4]. It has been postulated that derangements in this energy production process may play an integral role in the development of heart failure and may even be present before structural and functional sequalae of cardiac failure become apparent [4,5]. This has been most thoroughly investigated in HFrEF, whereby derangements in all three key steps of substrate utilisation, energy production, and energy transport have been shown. Moreover, impairment of myocardial energetics as assessed by phosphorous spectroscopy has been shown to correlate with higher symptom burden and worse prognosis in HFrEF [6].

Despite this clear link between HFrEF and myocardial energetics, the most energetically demanding part of the cardiac cycle is diastole, where ATP is used to break actomyosin cross links and thus allow relaxation [5]. Moreover, the most energetically demanding enzyme in the contractile apparatus, the sarcoplasmic reticular calcium ATPase (SERCA), will be affected early by any derangements in metabolism, preventing calcium lowering in diastole [7]. Therefore, whilst not as thoroughly investigated as HFrEF, it is unsurprising that patients with HFpEF show impaired myocardial energetics as shown by phosphorous spectroscopy. What is more, the individual risk factors associated with HFpEF, namely, hypertension, obesity, and diabetes, are all independently associated with impaired myocardial energetics. It is therefore clear that myocardial metabolism, at the very least, plays an important role in the pathogenesis of HFpEF.

## 2. Normal Cardiac Metabolism

**Figure 1:**
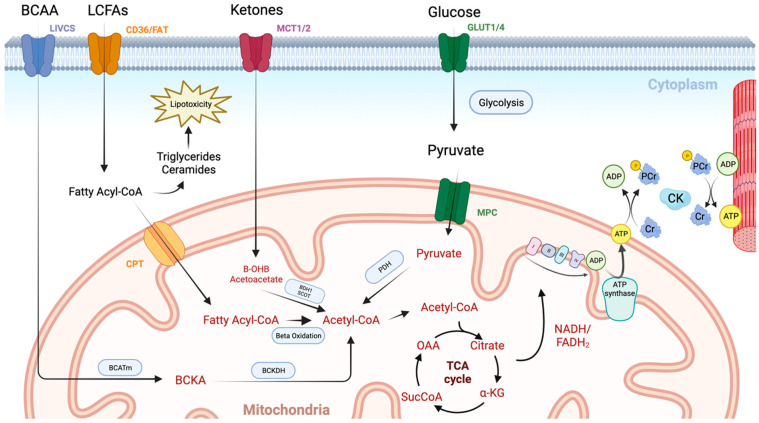
An overview of cardiac metabolism. Long chain fatty acids (LCFAs) enter the myocardium via CD36 and fatty acid transport protein (FAT). They then undergo esterification to form a fatty acyl coenzyme A (Acyl-CoA), which is either converted to lipotoxic intermediates such as triglycerides and ceramides or enters the mitochondrial via the carnitine palmitoyltransferase (CPT) system. Once inside the mitochondrial matrix, the fatty acyl-CoA undergoes beta oxidation with the resulting acetyl-CoA molecule entering the TCA cycle. Glucose enters the cardiomyocyte through glucose transporters (GLUT1/4). It then undergoes glycolysis producing pyruvate, which enters the mitochondria via the mitochondrial pyruvate carrier (MPC). Pyruvate dehydrogenase (PDH) governs the conversion of pyruvate into acetyl-CoA, and thus controls the balance between fatty acid and glucose use via the Randal cycle. Ketones are taken up via monocarboxylate transporters (MCT1/2) and are then converted to acetyl-CoA in the mitochondria by beta hydroxybutyrate dehydrogenase 1 (BDH1) and succinyl-CoA:3 oxoacid-CoA transferase (SCOT). Branched chain amino acids (BCCA) are taken up via the branched-chain amino acid–cation symporter family (LIVCS). Conversion to branched chain ketoacids (BCKA) occurs via the mitochondrial branched-chain aminotransferase (BCATm) before final conversion to acetyl-CoA via branched chain-alpha-ketoacid dehydrogenase (BCKDH). High-energy electron carriers (NADH and FADH_2_), produced via the tricarboxylic acid (TCA) cycle, deliver electrons to the electron transport chain, where they are used to generate a proton gradient across the inner mitochondrial membrane. ATP synthase then uses this proton-motive force to phosphorylate adenosine diphosphate (ADP) molecules, generating adenosine triphosphate (ATP). Transport of energy to the myofibril then occurs using the creatine kinase (CK) system. Image created using BioRender.com.

The initial steps in ATP generation are the uptake and utilisation of fuels in the myocardium. Under normal conditions, the heart sources the majority of its ATP from fatty acids (70–90%) and to a lesser extent carbohydrates (10–40%), with smaller contributions from ketone bodies and amino acids (Figure 1) [4]. The final end product of these pathways is acetyl coenzyme A which can then be fed into the tricarboxylic acid (TCA) cycle. The heart is metabolically flexible, adapting its substrate usage based on local and systemic conditions, allowing ATP generation to continue in fed, fasted, and high-demand states.

The second stage in this process is the generation of ATP via oxidative phosphorylation, which accounts for >95% of cardiac ATP. Following the generation of high-energy electron carriers (NADH and FADH_2_) in the TCA cycle, and to a lesser extent glycolysis, electrons are donated to the electron transport chain allowing the establishment of a proton gradient across the inner mitochondrial membrane. ATP synthase then utilises this gradient to phosphorylate adenosine diphosphate (ADP), creating ATP (Figure 1).

The final phase is ensuring the highly energetic ATP molecule can be transferred to the myofibrils for utilisation. Given its relatively large size and polarity, this is achieved via a phosphate transfer system. Creatine kinase catalyses the conversion of creatine to phosphocreatine (PCr) at the mitochondria, which can then diffuse freely to the myofibril (Figure 1). Here, the cytosolic isoenzyme catalyses the reverse reaction, allowing phosphorylation of the recently produced ADP, replenishing ATP supplies at the myofibril. Not only does this system function as a spatial energy shuttle, but it also serves an important temporal energy buffer, whereby PCr can replenish ATP supplies by phosphate donation at times of high demand.

## 3. Cardiac Metabolism in HFpEF

### 3.1. Animal Studies

Much of our knowledge of myocardial metabolism in heart failure comes from ischaemia–reperfusion animal models which reliably progress to HFrEF. However, given the distinct risk factors, pathophysiological mechanisms, and clinical phenotype in HFpEF, any derangement in cardiac metabolism may be different from than observed in HFrEF. Animal models of HFpEF have, however, proven more difficult to develop, partly owing to the heterogeneity in the clinical phenotype of HFpEF patients. Early studies focused on HFpEF risk factors such as obesity, hypertension, diabetes, and ageing, with animal models of these conditions in isolation being used as surrogates for HFpEF. Whilst many of these models showed classic features of HFpEF such as diastolic dysfunction and preserved systolic function, they often lacked key clinical features such as pulmonary congestion and elevated filling pressures [8].

More advanced animal models of HFpEF have sought to combine hypertensive, metabolic, and ageing stressors and have had more success in generating a more comprehensive set of HFpEF features. For instance, a novel ‘three-hit’ HFpEF mouse model has recently been developed, with obesity induced via a high-fat diet and hypertension via an injection of desoxycorticosterone pivalate (DOCP), with ageing as the final ‘hit’. This model accurately replicates key haemodynamic and clinical features of HFpEF including diastolic dysfunction, reduced exercise tolerance, and pulmonary congestion, all whilst maintaining left ventricular ejection fraction [9].

Interestingly, hypertensive and metabolic stresses appear to have competing consequences for substrate utilisation. In single-factor hypertensive models, a shift away from fatty acid oxidation towards glucose metabolism becomes apparent [10], similar to that seen in HFrEF. Whilst traditionally this shift was believed to confer an efficiency benefit in terms of oxygen use, it is becoming more established that this increased glycolytic activity occurs to support aspartate synthesis, a key intermediate in nucleotide synthesis, which is important in driving cardiac hypertrophy [11]. In contrast, metabolic models such as db/db mice [12,13,14], ZDF rats [15,16], and HFD/STZ rats [17,18] have increased fatty acid oxidation, presumably due to insulin resistance and decreased access to glucose. In the previously mentioned ‘three-hit’ HFpEF model, the authors found that fatty acid utilisation was slightly increased. It may be the case that the insulin resistance present caused a reciprocal increase in fatty acid oxidation via the Randal cycle, ultimately explaining the slightly increased fatty acid oxidation. It is clear, however, that there are complex pathways governing substrate use and that the flux of these pathways is likely to change with comorbidities and therefore HFpEF phenotype.

The increase in fatty acid oxidation does not appear to be sufficient to prevent accumulation of fatty acids and associated lipotoxicity. This intramyocardial lipid accumulation appears to be directly related to the development of diastolic dysfunction [19,20,21]. Fatty acids gain entry to the cytoplasm via the fatty acid translocase (CD36) located on the cytoplasmic membrane. Upon entry, they must cross the mitochondrial membrane in order to undergo fatty acid oxidation. Long-chain fatty acids, however, are unable to cross this membrane and require the aid of a carnitine shuttle. The key transporter in this shuttle is carnitine palmitoyltransferase 1 (CPT1), which regulates fatty acid uptake into the mitochondria and thus oxidative phosphorylation. In murine models, knockdown of CD36 decreased myocardial lipid accumulation and associated diastolic dysfunction [22], whilst CPT1 knockout mice showed increased lipid accumulation and cardiomyocyte apoptosis [23]. How this cytoplasmic accumulation of lipid species results in diastolic dysfunction has not been fully elucidated but is likely to involve reactive oxygen species (ROS) production [24,25,26], mitochondrial dysfunction [27,28], and disruption to calcium homeostasis [29].

Ketone bodies are an alternative myocardial fuel, and recent work has suggested that they contribute to myocardial metabolism in the healthy human heart [30]. These are synthesised from acetyl coenzyme A in hepatocytes and provide a carbon source for ATP synthesis in times of fasting, exercise, and exogenous ketone ingestion [31]. In HFrEF animal models, ketone body utilisation increases in what appears to be an adaptive response [32,33]. Furthermore, mice with cardio-specific knockdown of key ketolytic enzymes experienced worsening hypertrophy and systolic dysfunction [34], while infusion of ketone bodies appears to be protective [34,35]. However, in a HFpEF mouse model combining age, obesity, and hypertension, ketone body oxidation appeared to be reduced [9]. Interestingly, supplementation with beta-hydroxybutyrate ameliorated the HFpEF phenotype, although this effect appeared to be mediated via decreases in inflammation and mitochondrial dysfunction rather than provision of a myocardial energy substrate [9]. Furthermore, the study found that beta-hydroxybutyrate exhibited a dose-dependent activation of citrate synthase, the key regulator in governing flux of acetyl coenzyme A into the TCA cycle, and downregulated fatty acid uptake. This reduction in the intracellular acetyl-CoA pool appeared to inhibit mitochondrial acetylation, ultimately protecting against mitochondrial dysfunction [9].

Downstream of substrate utilisation, few studies have probed myocardial energetics in animal models of HFpEF [8]. Studies using 31-phosphorous magnetic resonance spectroscopy have shown decreases in PCr/ATP with obesity [36,37] and ageing [38] in murine models. However, data are lacking for phosphorous spectroscopy studies in two- and three-hit animal models of HFpEF. This is likely to be important in validating these newer models of HFpEF against human HFpEF.

Finally, ATP generated at the mitochondria must be transferred to the myofibrils via the creatine kinase (CK) shuttle. Again, this has been studied in conditions predisposing to HFpEF rather than in the condition directly. Forward CK flux ([phosphocreatine] x CK forward rate constant) has been shown to be significantly reduced in canine and porcine models of left ventricular hypertrophy (LVH) [39,40], with greater reductions present upon development of heart failure [39]. In isolated perfused hearts of obese mice who exhibited diastolic dysfunction, CK flux was maintained due to elevations in CK forward rate constant [37]. Similarly, in a diabetic rodent model, the CK forward rate constant was increased with CK flux similar to matched controls [41]. Future studies should seek to investigate CK kinetics in animal models of HFpEF that combine risk factors.

### 3.2. Human Studies

In contrast to the animal studies mentioned above, the bulk of work probing myocardial metabolism in human subjects with HFpEF has been directed at downstream ATP production. Most of this work has utilised phosphorous spectroscopy to non-invasively assess PCr/ATP ratio. Decreases in PCr/ATP in human subjects with HFrEF are well recognised [6,42,43,44], and PCr/ATP has been shown to be a better predictor of mortality than left ventricular ejection fraction [42]. Similarly, in HFpEF patients, a 20–27% reduction in PCr/ATP has been shown [45,46,47]. Moreover, PCr/ATP has been shown to be reduced in the hearts of patients with risk factors for HFpEF such as obesity [48,49], diabetes [45,50], hypertension [51], and ageing [52]. Interestingly, recent work has linked this energetic deficit in HFpEF to impaired left ventricular diastolic reserve, left atrial dilation, and pulmonary congestion during exercise, supporting the importance of energetic derangement as a key mechanism in HFpEF [45].

Once ATP is produced, the creatine kinase shuttle is crucial in the transportation of high-energy phosphates from mitochondria to myofibrils for utilisation. In patients with non-ischaemic cardiomyopathy, myocardial CK flux has been shown to predict heart failure outcomes independent of left ventricular ejection fraction and NYHA class [53]. Whilst limited data are available for CK flux in HFpEF, it has been studied in left ventricular hypertrophy (LVH) [54] and in obesity [48]. In hypertension-related LVH, the forward rate constant of myocardial CK reaction was normal, with CK flux reduced by 30% compared with healthy controls. However, in those with LVH and associated systolic dysfunction, the forward rate constant was halved and CK flux was reduced by almost two-thirds [54]. In obesity, the forward rate constant of the myocardial CK reaction at rest was found to be increased, yielding no overall difference in resting ATP delivery (CK flux) [48]. However, upon exercise, this flux was unable to be augmented, resulting in cardiopulmonary exercise intolerance [48]. Given exercise intolerance is a cardinal feature of HFpEF, it will be interesting to examine CK flux at rest and stress in individuals living with HFpEF.

Upstream substrate utilisation has not been fully assessed in patients living with HFpEF. Studies in patients living with obesity have shown correlations between insulin resistance and increased myocardial fatty acid uptake, utilisation, and oxidation [55], while studies in patients with diabetic cardiomyopathy have shown increased myocardial fatty acid uptake and oxidation and decreased glucose uptake [56,57]. A recent study investigated metabolomics in plasma and myocardial biopsies from HFrEF, HFpEF, and controls. Interestingly, myocardial but not plasma metabolites separated the groups, with both HFrEF and HFpEF patients having lower metabolites of fatty acid oxidation than controls [58]. Furthermore, ketones and branch chain amino acid metabolites were reduced in HFpEF patients, suggesting decreased fuel flexibility, and TCA intermediates were reduced, suggesting decreased anaplerosis. Whilst glucose metabolism was not fully assessed, HFpEF patients had higher levels of pyruvate than controls and decreased gene expression of multiple proteins central to glucose metabolism. HFpEF may therefore be characterised by failure to utilise fatty acids, which the myocardium has become dependent on due to relevant comorbidities such as obesity and diabetes.

Until recently, most information gained has been a snapshot of myocardial substrate use at a single point in time. The development of hyperpolarised ^13^C magnetic resonance spectroscopy, however, allows real-time visualisation of substrate uptake and metabolism in the myocardium [59]. Using hyperpolarized [1-^13^C]pyruvate, pyruvate dehydrogenase (PDH) flux can be obtained and therefore the balance between carbohydrate and fatty acid usage estimated. This technique has been used in those living with diabetes, showing decreased cardiac PDH flux accompanied by reduced myocardial energetics [60]. This decrease in PDH flux is consistent with a myocardium that has become reliant on fats in the face of insulin resistance. Future studies should seek to apply this technique to HFpEF.

A mismatch between the uptake and oxidation of fatty acid species leads to their accumulation and resultant lipotoxicity. Proton (^1^H) spectroscopy can be used to non-invasively assess myocardial triglyceride content and has been used to show that HFpEF patients have significantly more intramyocardial fat than either HFrEF or control subjects [61]. Furthermore, intramyocardial fat correlated with the severity of diastolic dysfunction independently of risk factors. The extent to which myocardial lipotoxicity drives HFpEF is unknown, but it represents an attractive treatment target.

## 4. Therapeutic Strategies

Given the metabolic derangements in HFpEF discussed above, therapeutic modulation of metabolic pathways represents an exciting new treatment strategy for HFpEF. This may involve manipulating fatty acid, glucose, or ketone oxidation (Table 1) to yield improved ‘fuel efficiency’, improving contractile function by increased ATP availability.

### 4.1. Balancing Fatty Acid Metabolism

Balancing myocardial fatty acid uptake and oxidation to reduce lipotoxicity and improve bioenergetics represents a viable strategy for the treatment of HFpEF and may be particularly beneficial in the cardiometabolic phenotype.

Therapies have largely sought to achieve this balance by reducing circulating fatty acids and/or decreasing fatty acid uptake. Perhaps the most intuitive method of achieving this is through weight loss. Remarkably, the effect of weight loss on myocardial energetics in HFpEF is yet to be studied. However, weight loss in people living with obesity without heart failure leads to reductions in CK flux [48] and increased PCr/ATP [62]. Moreover, dietary weight loss has been shown to reduce myocardial steatosis and improve diastolic function in patients living with obesity and diabetes [63]. Interestingly, weight loss induced by bariatric surgery does not appear to decrease myocardial steatosis [89].

Circulating fatty acid levels can be reduced by preventing their release from adipocytes using the lipolysis inhibitor, niacin (nicotinic acid) [90]. No studies have assessed the utility of niacin in heart failure. However, acipimox, a niacin derivative, has been associated with either no change [64] or decreases in cardiac performance and efficiency [65] in HFrEF models.

Peroxisome proliferator-activated receptor (PPAR) agonists also have the ability to reduce circulating lipids. To date, no clinical studies have prospectively investigated PPAR agonists in HF. However, a post hoc analysis from the Action to Control Cardiovascular Risk in Diabetes (ACCORD) trial, revealed that the PPAR agonist, fenofibrate, was effective in reducing heart failure hospitalisations [66]. Further prospective studies of PPAR agonists are now warranted.

Emerging therapeutic targets of fatty acid uptake include the oral small molecule SIRT6 activator, MDL-800. SIRT6 acts as an endothelial gate-keeper, limiting fatty acid uptake, and appears to be reduced under diabetic conditions. Overexpression of SIRT6 in diabetic conditions protects against HFpEF. A recent study has shown that MDL-800 protected against cardiac lipid accumulation and diastolic dysfunction in a diabetic mouse model [18]. Further studies of MDL-800 and, indeed, therapies which target endothelial fatty acid transport are needed.

Whilst targeting the renin–angiotensin–aldosterone system (RAAS) has previously failed to improve outcomes in HFpEF patients [91,92], novel preclinical work has shown promise in targeting specific aspects of the pathway [13]. In a db/db diabetic mouse model of diastolic dysfunction, administration of angiotensin 1–7, the product of angiotensin converting enzyme 2 (ACE2) and a negative regulator of the RAAS, improved diastolic function and reduced myocardial lipid accumulation [13]. Decreases in cardiac triacylglycerol and ceramide levels occurred concomitantly with increases in myocardial lipase expression, which correlated with increased levels of SIRT1 and deacetylation of FOXO1 [13]. Indeed, cardiomyocyte steatosis in HFpEF is associated with increased activity of FOXO1, which when depleted ameliorates the HFpEF phenotype in mice and reduces myocardial lipid accumulation [19].

Conversely, stimulation of fatty acid β-oxidation has been trialled, aiming to increase cardiac energy production, mitochondrial function, and the efficiency of SERCA in HF. In pressure overload-induced HF mice, metabolism was switched from glycolysis to fatty acid β-oxidation following administration of astragaloside IV [67]. This led to improved myocardial function and increased expression of PPARα and SERCA2a, associated with increased ATP production and enhanced mitochondrial function [67]. In a rodent model of metabolic syndrome, astragaloside IV improved diastolic dysfunction [68]. This appeared to be associated with reductions in oxidative stress and circulating lipids, pointing towards a link with myocardial lipid metabolism. Additional studies will be required to investigate the effect of astragaloside IV on lipid metabolism in HFpEF.

Given the key role of the carnitine palmitoyltransferase shuttle in transporting fatty acids into the mitochondria, supplementation of L-carnitine has been proposed to increase myocardial fatty acid usage. Administration of L-carnitine in a rodent hypertensive HFpEF model restored depleted LV free carnitine, which consequently attenuated LV fibrosis and pulmonary congestion, improving diastolic function and survival [69]. L-carnitine treatment was also shown to reduce diastolic dysfunction and improve symptoms in a small blinded placebo-controlled trial in patients with diastolic heart failure [70].

Hyperacetylation of key enzymes in the fatty acid oxidation pathway was identified in the HFpEF mouse heart, and correlated with reduced NAD+/NADH ratio, impaired mitochondrial function, depleted TCA cycle metabolites [93,94], and downregulation of SIRT3 [94]. NAD+ repletion by supplementation of nicotinamide riboside (NR) downregulated the acetylation level, improved mitochondrial function, and ameliorated HFpEF phenotypes [93,94]. Indeed, use of the mitochondria-targeted antioxidant, Mito-TEMP, increased mitochondrial antioxidants and stimulation of fatty acid metabolism and improvements in cardiovascular function in spontaneously hypertensive rats [71]. In a two-hit mouse model of HFpEF with impaired nitric oxide (NO) bioavailability, mitochondrial respiration, aortic vascular function, and exercise capacity, treatment with sodium nitrite and hydralazine restored NO bioavailability, reduced oxidative and nitrosative stress, preserved endothelial function and mitochondrial respiration, limited the fibrotic response, and improved exercise capacity [95].

Historically in the HFrEF heart, metabolism has been said to be inflexible, with a shift away from fatty acid metabolism towards glycolytic metabolism, uncoupling of glycolysis, and glucose oxidation. Recent work, however, has called this inflexibility into question. An intralipid infusion in non-ischaemic HFrEF patients increased myocardial fatty acid uptake and oxidation and resulted in increases in PCr/ATP and LV systolic function [72]. Interestingly, myocardial energetics were better with the intralipid infusion compared with an insulin–glucose infusion, suggesting that these HFrEF hearts performed better when fatty acid metabolism was augmented. Whether HFpEF hearts are metabolically flexible is yet to be studied but warrants further investigation. It should be noted, however, that further delivery of lipids to a heart already suffering from myocardial steatosis may exacerbate lipotoxicity.

### 4.2. Increasing Glucose Utilisation

Improving myocardial glucose utilisation represents another treatment strategy to improve cardiometabolic efficiency. This can be achieved by leveraging the Randle cycle, where decreases in fatty acid oxidation-derived acetyl coenzyme A promote increased PDH flux, resulting in increased glucose oxidation [96,97]. The final enzyme in fatty acid oxidation, 3 ketoacyl-CoA thiolase, can be inhibited with trimetazidine. Trimetazidine reduces the frequency of angina symptoms and improves exercise tolerance [98] and has been suggested to be beneficial in HFrEF [73,74], with a small randomised control trial, DoPING-HFpEF, ongoing [75]. Ranolazine, the anti-anginal with late sodium channel inhibitory properties, also has the ability to partially inhibit fatty acid oxidation, thus increasing glucose utilisation via the Randle cycle [99]. In a small randomised trial of patients with HFpEF (RALI-DHF), ranolazine improved haemodynamics (pulmonary capillary wedge pressure and LV end diastolic pressure) but did not change relaxation parameters [76].

Inhibition of CPT1, responsible for transport of fatty acids into the mitochondria matrix, has been investigated as a potential therapeutic target to theoretically increase glucose utilisation. Use of the CPT1 inhibitor etomoxir was demonstrated to improve contractile function in a rodent model of afterload-dependent hypertrophy [100], improving the rate of SERCA-dependent calcium reuptake [101]. In a small, open-label study of extomoxir in 10 patients with HFrEF, there was a modest improvement in cardiac function [77]. However, generic inhibitors of CPT1 have raised concerns with off-target neurotoxicity and hepatotoxicity. Use of perhexiline, a cardiac-selective CPT1 inhibitor, has been associated with increased PCr/ATP ratio in patients with hypertrophic cardiomyopathy, with concomitant improvement in symptomology assessed with NYHA score [78]. This was observed in the absence of change in LVEF or cardiac metabolic substrate utilisation. A randomised controlled trial of perhexiline in 70 patients with HFpEF was completed in 2014 but the data remain unpublished (NCT00839228).

Finally, a novel partial fatty acid oxidation inhibitor, ninerafaxstat, represents an emerging treatment strategy for HFpEF and is thought to increase myocardial metabolic efficiency by altering myocardial substrate utilisation to favour glucose oxidation. IMPROVE-DiCE (NCT04826159) Part 1 enrolled patients with type 2 diabetes and obesity, and results demonstrated improvement of cardiac energetics with significantly improved cardiac steatosis and diastolic filling [79]. Moreover, the use of hyperpolarized magnetic resonance spectroscopy showed increases in PDH flux, supporting the proposed mechanism of substrate switching. This is being expanded to patients with cardiometabolic HFpEF in Part 2 of IMPROVE-DiCE, which will assess the impact of ninerafaxstat on cardiac energetics, diastolic function, functional capacity, and heart failure symptoms.

Manipulation of glucose metabolism may also occur with administration of dichloroacetate (DCA), a pyruvate dehydrogenase kinase inhibitor. This results in increased flux through PDH and thus linking of glycolysis with glucose oxidation. Whilst it does not appear beneficial in HFrEF [102], in preclinical models of HFpEF, DCA has been demonstrated to improve myocardial contractility, decrease hypertrophy, and improve animal survival [80,81]. This is underpinned by increased energy reserves and glucose uptake through activation of the pentose phosphate pathway alongside reduced oxidative stress [80,81]. Concerns remain regarding the neurotoxicity of DCA; however, research is ongoing whether this is mitigated by antioxidants or muscarinic receptor antagonists [103].

Glucose metabolism may be enhanced with the glucagon-like peptide-1 receptor agonists (GLP1-RA), which aim to increase insulin secretion, sensitivity, and glucose uptake. Preclinical studies have confirmed increased glucose metabolism with GLP1-RA [104] and improvements in LV function in HFrEF [105]. In a murine HFpEF model, the GLP1-RA liraglutide attenuated cardiometabolic dysregulation and improved cardiac function to a greater extent than the sodium–glucose transporter 2 inhibitor (SGLT2i), dapagliflozin [82]. The recently published STEP-HFpEF trial showed the benefit of the GLP1-RA semaglutide in inducing weight loss and improving symptoms in patients with HFpEF and obesity [106]. Studies investigating the effect of GLP1-RA on cardiac metabolism in humans are awaited, but early results suggest improvements in cardiac energetics [83].

### 4.3. Increasing Ketone Body Utilisation

A switch to ketone metabolism represents a potential alternative source of fuel for the myocardium. This would be a more energetically efficient fuel source, requiring fewer moles of oxygen per moles of ATP produced, and reducing over-reliance on fatty acids [5,107]. However, given the reciprocal relationship between ketone body usage and fatty acid and glucose metabolism, increasing ketone body usage may ultimately have an unfavourable effect on myocardial energetics.

Recent preclinical data suggest HFpEF pathogenesis may be ameliorated by promoting β-hydroxybutyrate abundance, where β-hydroxybutyrate antagonises proinflammatory cytokine-triggered mitochondrial dysfunction and fibrosis in murine HFpEF [9]. A further murine study found that β-hydroxybutyrate improved diastolic function, fibrosis, and cardiac inflammation through increased Treg cells [84]. Supplementation of ketone esters was found to reverse adverse cardiac remodelling and enhance ventricular function in murine and rodent heart failure models [35]. Clinical studies increasing ketone body usage in the human HFpEF heart are awaited, but studies of ketone body infusion in HFrEF patients have yielded beneficial haemodynamic effects [85].

However, negative effects of ketogenic diets have also been observed. Ketogenic diets, frequent prolonged fasting, or exogenous β-hydroxybutyrate administration in healthy rodents reduced mitochondrial biogenesis and cell respiration and increased cardiac apoptosis/fibrosis [108]. This was also observed in human tissue samples, where increased β-hydroxybutyrate levels were associated with decreased mitochondrial biogenesis and increased cardiac fibrosis [108]. This was found to be related to acetylation of the Sirt7 promoter and activated Sirt7 transcription, which inhibited transcription of mitochondrial ribosome-encoding genes resulting in cardiac apoptosis/fibrosis [108]. Whilst an alternate-day ketogenic diet (with a medium-chain triglyceride) has been demonstrated to have a cardioprotective effect, a continuous ketogenic diet worsened diastolic function [109]. In vitro, ketone body supplementation limits phenylephrine-induced hypertrophy, partially by suppressed mTOR signalling [110]. Further, in spontaneously hypertensive rats, a ketogenic diet increased interstitial fibrosis and cardiac remodelling through mTOR signalling pathways [111].

SGLT2i, the only drug class to demonstrate a prognostic benefit in HFpEF patients [2,3,112], may exert their cardioprotective effects, in part, by increasing myocardial ketone body usage. Studies have shown that SGLT2i increase circulating ketone body levels [113,114,115]. In a porcine HFrEF model, empagliflozin led to a myocardial switch away from glucose towards ketone bodies and fatty acids, and this was associated with improved myocardial energetics and enhanced LV function [116]. Studies using cardiac magnetic resonance spectroscopy to assess myocardial PCr/ATP following SGLT2i treatment in patients living with type 2 diabetes have yielded conflicting results [86,87]. The EMPA-VISION trial, a randomized control trial of 72 HF patients, found empagliflozin treatment did not change cardiac energetics in HFrEF or HFpEF at rest or during dobutamine stress [88]. Similarly, serum metabolomics and circulating ketone bodies were unchanged [88]. Further work is needed to assess the cardioprotective mechanisms of SGLT2i and whether they are able to alter myocardial substrate utilisation and thus myocardial metabolism.

## 5. Conclusions

Metabolic disturbances play a crucial role in heart failure with preserved ejection fraction (HFpEF), presenting a unique opportunity for both understanding and treating this condition. Cardiometabolic risk factors, notably obesity and diabetes, significantly impact cardiac substrate utilisation. This leads to reduced myocardial ATP production and subsequent diastolic dysfunction. The insulin resistance triggered by these comorbidities tends to diminish glucose utilisation, resulting in an increased reliance on fatty acids as a primary fuel source. The progression from risk factor to HFpEF may be marked by the heart’s diminished capacity to increase fatty acid metabolism, creating a mismatch between supply and utilisation. This imbalance can lead to myocardial steatosis and lipotoxicity. Consequently, targeting myocardial metabolism emerges as a promising approach in HFpEF treatment. Preliminary studies in animal models and human HFpEF patients have begun to explore ways to modify and enhance metabolism of fatty acids, glucose, and ketone bodies. However, further extensive research is essential to fully decipher the metabolic alterations in HFpEF and to uncover novel therapeutic targets.

## Figures and Tables

**Table 1:** Therapeutic strategies targeting myocardial metabolism in HFpEF.

Therapeutic Approach	Rationale	Summary	References
***Balancing Fatty Acid Metabolism***			
Weight Loss	Reduction in circulating lipids and myocardial steatosis.	Not yet studied in HFpEF, but improved myocardial energetics in obesity +/− diabetes and reductions in myocardial steatosis following dietary weight loss.	[62,63]
Nicotinic acid derivates	Reduction in circulating lipids.	No studies of niacin in HF, but acipimox (niacin derivative) has shown disappointing results in HFrEF.	[64,65]
PPAR agonists (e.g., fenofibrate)	Reduction in circulating lipids.	No prospective studies, but post hoc analysis suggests reduced HF hospitalisations.	[66]
SIRT6 activator (MDL-800)	Reduction of fatty acid translocation across the endothelium.	Protection against cardiac steatosis and diastolic function in murine model.	[18]
Angiotensin 1–7	Negative regulator of RAAS.	Improved diastology and reduced myocardial steatosis with increase in cardiac lipase expression in a diabetic murine model.	[13]
Astragaloside IV	Stimulation of fatty acid β-oxidation.	Switch from glucose to fatty acid oxidation, with improved energetics and function in murine HFrEF model. Improved diastology in HFpEF rodent model.	[67,68]
L-carnitine	Increase fatty acid transport into the mitochondria via carnitine shuttle.	Improvements in diastology, pulmonary congestion, and survival in hypertensive HFpEF rodent model. Reduction in symptoms and diastolic dysfunction in diastolic HF patients.	[69,70]
Nicotinamide riboside	NAD+ repletion, decreasing acetylation of key enzymes in FAO pathway.	Improved mitochondrial function and symptoms in HFpEF murine model.	[71]
Intralipid infusion	Supplying myocardium with a rich fuel source which it prefers in health.	Studied only in HFrEF patients, but improvements in energy production and LV systolic function.	[72]
***Improving Glucose Utilisation***			
Trimetazidine	Inhibition of FAO in order to increase glucose oxidation.	Some suggested benefit in HFrEF, ongoing DoPING-HFpEF study will evaluate in HFpEF.	[73,74,75]
Ranolazine	Inhibition of FAO in order to increase glucose oxidation.	Improved haemodynamics in HFpEF patients with no change in relaxation parameters (RALI-DHF).	[76]
Etomoxir	Inhibiting CPT1, responsible for transport of fatty acids into the mitochondria.	Improvement in function in 10 HFrEF patients, no studies in HFpEF, limited by neurotoxicity and hepatotoxicity.	[77]
Perhexilline	Inhibiting CPT1, responsible for transport of fatty acids into the mitochondria.	Improvements in symptoms and myocardial energetics in HCM. RCT in HFpEF completed but not reported (NCT00839228).	[78]
Ninerafaxstat	Inhibition of FAO in order to increase glucose oxidation.	Normalisation of myocardial energetics and improved diastolic filling and cardiac steatosis in diabetic cardiomyopathy.	[79]
Dichloroacetate	Inhibition of PDHK, thus increasing PDH flux and linking glycolysis with glucose oxidation.	Preclinical HFpEF models show improvements in contractility, hypertrophy, and increased energy reserves. Concerns over neurotoxicity.	[80,81]
GLP1-RA	Increased insulin secretion and sensitivity, allowing increased glucose uptake.	Improvements in cardiac function greater than SGLT2i in murine HFpEF model. Early results in humans hint at improvements in cardiac energetics.	[82,83]
***Increasing Ketone Body Utilisation***			
Ketone body supplementation	Alternative fuel source requiring less oxygen per mole of ATP produced.	Beneficial effects in human HFrEF and potential benefits in animal HFpEF models, clinical studies in HFpEF are awaited.	[9,35,84,85]
SGLT2i	Shown to increase circulating ketone body levels, offering alternative myocardial fuel.	Conflicting results regarding changes in myocardial energetics in HFpEF with SGLT2i treatment. Further work is needed.	[86,87,88]

HF: heart failure, HFpEF: heart failure with preserved ejection fraction, HFrEF: heart failure with reduced ejection fraction, PPAR: peroxisome proliferator-activated receptors, SIRT6: sirtuin 6, RAAS: renin–angiotensin–aldosterone system, FAO: fatty acid oxidation, CPT1: carnitine palmitoyltransferase 1, RCT: randomised controlled trial, PDHK: pyruvate dehydrogenase kinase, GLP1-RA: glucagon-like peptide-1 receptor agonist, SGLT2: sodium glucose transporter 2, ATP, adenosine triphosphate.

## Data Availability

Not applicable.
